# Pharmaceutical Discharge Management: Implementation in Swiss Hospitals Compared to International Guidelines

**DOI:** 10.3390/pharmacy9010033

**Published:** 2021-02-07

**Authors:** Helene Studer, Fabienne Boeni, Kurt E. Hersberger, Markus L. Lampert

**Affiliations:** 1 Pharmaceutical Care Research Group, Department of Pharmaceutical Sciences, Faculty of Science, University of Basel, 4051 Basel, Switzerland; fabienne.boeni@unibas.ch (F.B.); kurt.hersberger@unibas.ch (K.E.H.); markus.lampert@unibas.ch (M.L.L.); 2 Clinical Pharmacy, Institute of Hospital Pharmacy, Solothurner Spitäler AG, 4600 Olten, Switzerland

**Keywords:** medication management, hospital discharge, clinical pharmacy, pharmaceutical care, seamless care, hospital pharmacy services, patient-related activities, survey, Switzerland

## Abstract

Readmissions to the hospital are frequent after hospital discharge. Pharmacist-led interventions have been shown to reduce readmissions. The objective of this study was to describe pharmacist-led interventions to support patients’ medication management at hospital discharge in Switzerland and to compare them to international guidelines. We conducted a national online survey among chief hospital pharmacists focusing on medication management at hospital discharge. To put our findings in perspective, Cochrane reviews and guidelines were searched for summarised evidence and recommendations on interventions. Based on answers in the survey, hospitals with implemented models to support patients at discharge were selected for in-depth interviews. In semi-structured interviews, they were asked to describe pharmacists’ involvement in the patients’ pathway throughout the hospital stay. In Swiss hospitals (*n* = 44 survey participants), interventions to support patients at discharge were frequently implemented, mostly “patient education” (*n* = 40) and “communication to primary care provider” (*n* = 34). These interventions were commonly recommended in guidelines. Overall, pharmacists were rarely involved in the interventions on a regular basis. When pharmacists were involved, the services were provided by hospital pharmacies or collaborating community pharmacies. In conclusion, interventions recommended in guidelines were frequently implemented in Swiss hospitals, however pharmacists were rarely involved.

## 1. Introduction

After hospital discharge, patients are at risk for unplanned readmissions. In a large US study, including over 31 million index hospital admissions, the rate of unplanned readmissions was 11.6% [1]. A systematic review including 19 studies found that a median of 21% of readmissions are drug-related, of which a median of 69% were considered to be preventable [2]. Drug-related problems (DRPs) occur frequently after hospital discharge. Reported problems include non-adherence, inadequate dosing/duration/frequency/administration, and suboptimal drugs [3]. Medication errors occur in up to 50% of patients within 30 days of discharge [4].

Different attempts have been made to reduce hospital readmissions. It has been shown that pharmacists’ interventions such as medication reconciliation or patient education can reduce hospital readmissions, adverse drug event-related hospital revisits, DRPs, and emergency room visits after discharge [5,6,7]. Topics of discharge medication counselling include discussing dose and dosage, providing paper-based medication lists, explaining the indication of medicines, and/or discussing adverse drug reactions [8,9]. Studies showed that medication counselling at hospital discharge was more effective where it was part of a set of different interventions (e.g., combined with medication review, medication reconciliation, or telephone follow-up) rather than as a single intervention [9]. According to a systematic review, interventions bridging transitions of care can reduce hospital readmissions, these interventions include self-management education, telephone follow-up, and medication reconciliation [10]. Another systematic review found that complex interventions were associated with a reduction in relative risk of readmissions of 37%. This systematic review showed that interventions aimed at enhancing patient capacity for post-discharge care were effective in reducing the relative risk of readmission [11].

A survey conducted in Swiss hospital pharmacies found an average of 1.12 full-time equivalents of all employed pharmacists per 100 beds, of which 20% were allocated to clinical pharmacy services [12]. In Switzerland, a hospital pharmacy is an institution that is part of the hospital, managed by a registered pharmacist and offering pharmaceutical services to the hospital’s patients; it is not publicly accessible [13]. Hospital pharmacists carry out the following activities: management of pharmaceutical products, compounding of medicines in small quantities, and patient-oriented pharmacy/clinical services [14]. For specialization, there is a postgraduate degree in hospital pharmacy and a postgraduate certificate of proficiency in clinical pharmacy. The latter enables pharmacists to conduct “direct patient oriented pharmaceutical activities, developed on patient care wards in collaboration with other healthcare professionals” [14,15]. However, little is known on how the above-mentioned resources in Swiss hospitals are used to support patients at discharge. In Switzerland, patients are normally discharged without contacting the hospital pharmacy and without medication to take home. Patients receive a discharge prescription that can be filled in a community pharmacy [16].

The aim of this study was to describe the frequencies of different interventions to support patients’ medication management at discharge in Swiss hospitals. We also aimed to describe different pharmacist-led models that were implemented. As there are no official guidelines on this topic in Switzerland, we compared our findings to international guidelines.

## 2. Materials and Methods

### 2.1. Data Collection

We conducted an online survey among Swiss hospital pharmacies. The survey consisted of two parts. The first part described the development of clinical pharmacy services in Swiss hospitals and was published separately [12]. The second part focused on interventions to improve medication management at hospital discharge. To formulate questions regarding hospital discharge, literature was searched for existing discharge processes and models [17,18,19,20,21,22]. To verify the understandability of the questions, the survey was piloted with three German-speaking and one French-speaking practicing clinical pharmacists. The final version contained 73 questions. The interventions that were inquired by the questionnaire are summarized in Table 1. All chief hospital pharmacists registered at the Swiss Society of Public Health Administration and Hospital Pharmacists (GSASA) were asked by e-mail to participate (*n* = 60). The questionnaire was accessible from the beginning of June to the beginning of September 2017. Further details were published elsewhere [12].

To put the survey results in perspective, Cochrane reviews and international guidelines were searched for summarized evidence about the efficacy and recommendations of these interventions. We searched the Cochrane library for Cochrane reviews that evaluated interventions at hospital discharge or at transitions of care. The search string combined Medical Subject Headings (MeSH) with key search terms (see Appendix A). To find guidelines containing relevant recommendations usually published in grey literature sources, we conducted advanced Google searches using key search terms (see Appendix B). From these searches, we selected Cochrane reviews/guidelines from English-speaking countries that contained recommendations for healthcare professionals on medication management at the transition from hospital to home including every age group and excluding guidelines that were medical-condition-specific or specific to a single hospital. Two authors (FB, HS) independently screened the results of the Cochrane search and selected the relevant reviews based on the aforementioned criteria. Equally, the first 100 search results of the Google searches were screened by both authors and relevant guidelines were selected. Discrepancies were resolved by discussion. Evidence and relevant recommendations concerning the above-mentioned interventions were extracted from the selected full texts.

In order to depict in greater detail the different models implemented in Swiss hospitals to support patients in their medication management at hospital discharge, we conducted semi-structured, face-to-face interviews. Hospitals were eligible for these interviews if they indicated, in the survey, to have such a model in place. We selected hospitals with different models to support patients at discharge to present illustrative examples. In the interviews, pharmacists were asked to describe the pharmacists’ involvement in the patients’ pathways throughout their hospital stays. If the hospital collaborated with a community pharmacy (owned or unowned by the hospital) at discharge, they were asked to describe this collaboration. We defined a hospital community pharmacy as a pharmacy open to patients discharged from the hospital with full access to the hospital’s patient records, in contrast to regular Swiss community pharmacies, which do not have access to clinical data. The interviews were audiotaped and afterwards transcribed using MAXQDA 2020 (VERBI Software GmbH, Berlin, Germany). The transcripts were validated by a second researcher.

### 2.2. Data Analysis

Descriptive statistics were used to report the results of the survey. Continuous variables were expressed as a median with 25th and 75th percentiles (p25, p75) or as a sum and categorical variables as frequencies. Pharmaceutical interventions discussed during the interviews were summarized using the taxonomy of Hansen et al. [17] or commonly used terms (e.g., interprofessional ward round). Less common interventions were further detailed as text.

## 3. Results

### 3.1. Survey

A total of 44 hospital pharmacies participated in the survey (return rate = 73.3%). The pharmacists of all five Swiss university hospitals answered the questionnaire, as well as 18 cantonal or regional hospitals, 11 private hospitals or specialized clinics, and 10 hospitals that were organized in networks. They supplied a median of 340 beds (p25 = 200, p75 = 937.5, minimum = 82, maximum = 2000) and employed a total of 265.8 full-time equivalents of hospital pharmacists.

#### 3.1.1. Interventions Conducted by Healthcare Professionals to Support Patients at Hospital Discharge

The participating hospitals implemented a variety of pre- and post-discharge interventions involving healthcare professionals (e.g., physicians, pharmacists, and nurses) to support patients at hospital discharge (Table 2). Some participants did not know if the interventions were implemented, whereas others reported that the interventions were not implemented in their hospital.

The evidence promoting the implementation of these interventions and the guidelines recommending the interventions are listed in Table 2. The Cochrane search yielded 444 reviews, and, after the title and abstract screening, three Cochrane reviews [23,24,25] were selected according to the inclusion criteria. The Google search resulted in 16 guidelines [26,27,28,29,30,31,32,33,34,35,36,37,38,39,40,41].

In one hospital, pharmacists were involved in following patient education interventions on a daily basis: patient counselling on their medication, in-depth patient counselling on their medication, patient instructions and patient counselling on what to do with medication prescribed before hospital admission. Three hospital pharmacies were involved in patient education interventions several times a month or less. In two hospitals, pharmacists were involved in the communication of medication changes to at least one primary care provider on a daily basis. In five hospitals, pharmacists were involved less frequently.

Of the 44 hospital pharmacies, 29 indicated that there are guidelines in place for the process of hospital discharge, 4 answered that there are no such guidelines, and 11 did not know. In nine hospitals, there were efforts to identify patients in need of more intensive support at discharge. In 21 hospitals, they did not stratify patients and in 14 cases participants did not know. For the identification, the hospitals used inclusion criteria (*n* = 3), scores (*n* = 2), or specific conditions (*n* = 1). The services delivered to the selected patients consisted of: organizing aftercare (*n* = 3), organization of medicines (*n* = 3), discharge planning/coordination (*n* = 2), patient education (*n* = 2), and medication review (*n* = 1).

#### 3.1.2. Collaboration with a Community Pharmacy

**Figure 1:**
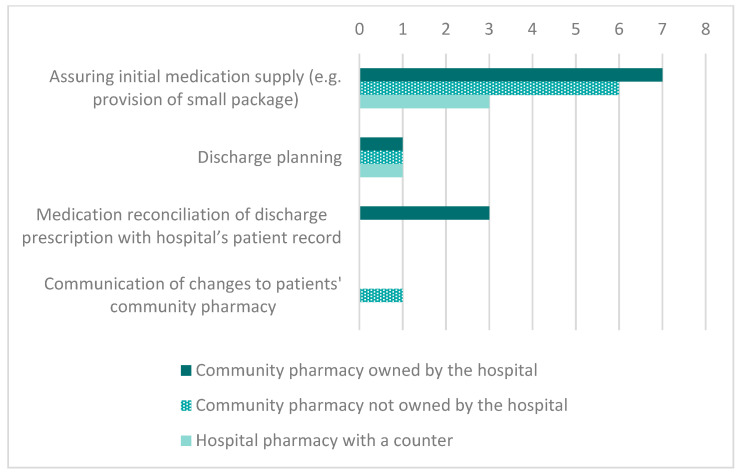
Frequency of predefined roles of the pharmacies that were collaborating with hospitals and that were accessible to patients at hospital discharge (multiple answers were possible), (*n* = 17).

Of the 44 participating hospitals, 17 (38.6%) collaborated with a community pharmacy (owned or unowned by the hospital) or had a counter in the hospital pharmacy that was open to discharged patients, and, of these, six had full access to the hospital’s patient records. The frequency of predefined roles of these 17 pharmacies is depicted in Figure 1. Additionally, “obtaining specific medications from the hospital pharmacy that are not easily available otherwise” and “conducting medication reviews and making adaptions to discharge prescriptions after consultation with the physician” was mentioned as a role by one pharmacy each.

Participants were asked to give a short description of the collaboration between the hospital pharmacy and the community pharmacy or the counter in the hospital pharmacy. Eleven of the 17 gave the following descriptions of the collaboration: in seven hospitals, the collaboration focused on logistic support (e.g., exchange of medication, provision of medicines at discharge), four hospitals had a close collaboration (but no further details were provided), and, in one hospital pharmacy, pharmacists answered general and patient-specific questions regarding the discharge prescription. Seven of the 17 pharmacies were contacted prior to patient discharges. Six of them received the discharge prescription in advance, one received a written report, and one was contacted in advance when there was a special order or when the patient needed an extemporaneous product (multiple answers were possible).

### 3.2. Interviews

Six hospitals that had a model implemented to support patients at discharge were contacted for an interview and all of them accepted. If they collaborated with a community pharmacy, the community pharmacy was also asked for an interview. In three out of four community pharmacies, pharmacists worked in the hospital pharmacy as well as in the community pharmacy. Table 3 gives an overview of the models implemented to support the patients’ pathways throughout the stays in these hospitals.

A **medication self-management training program** was implemented in one long-term rehabilitation clinic. Patients with long lengths of stay were assessed for suitability to participate. This program aimed at promoting patients’ independence and self-care. Starting two to three months before discharge, patients learned to manage their own medication. They received a prescription that they filled in the hospital community pharmacy. There the prescription was reconciled with the hospital’s patient record and the medication was dispensed. At the beginning, patients were supervised by a nurse when they prepared their medication, often using a pillbox, to check the patients’ capability of medication self-management. The hospital community pharmacy checked the patients’ adherence using dispensing records. If the pharmacists detected or suspected an adherence problem, they either talked to the patient directly or informed the responsible nurses.

In one hospital, **medication reviews** of discharge prescriptions were conducted **in the hospital pharmacy**. At hospital discharge, physicians sent electronic discharge prescriptions to the hospital pharmacy. There pharmacists performed a medication reconciliation and a medication review. Any issues detected were communicated to physicians. Once all issues were resolved, pharmacists approved the discharge prescriptions, which were then given to patients to fill in a community pharmacy. If patients were discharged before a pharmacist approved the discharge prescription, issues were still communicated to the physician. In case of an urgent issue, the patient was contacted by the physician or a coach; for less urgent issues, a comment was added in the discharge letter to general practitioners.

Of the interviewed hospitals, four had a **hospital community pharmacy**, either within the hospital or close by. At discharge, patients had the choice to fill their discharge prescription there or to take it to any regular community pharmacy. If filled in the hospital community pharmacy, prescriptions were sent there in advance. In one hospital, all prescriptions were sent to the hospital community pharmacy and patients had the choice to either collect their medication there or to take their prescription to another community pharmacy. Hospital community pharmacies performed medication reconciliation. One hospital community pharmacy additionally conducted medication reviews, using risk factors to stratify the depth of the medication review. In case of DRPs and discrepancies that needed clarification, they were communicated to the physician or the nurse and sometimes resulted in changes of discharge prescriptions. At discharge, patients were counselled on their medications; the depth of the counselling depended on the complexity of the prescription. If patients had a planned discharge outside of opening hours of the hospital community pharmacies, the medication was usually prepared in advance and picked up by a nurse or by the patient before discharge. However, if the discharge was unplanned, patients received the discharge prescription to fill in a regular community pharmacy. The hospital community pharmacies did not usually contact patients’ community pharmacies, except for one that only dispensed small packages and forwarded prescriptions to the patients’ community pharmacies, requesting them to follow-up on patients within a few days after discharge. The forwarded prescription contained a quick response (QR) code to reduce the workload of following institutions and to help trace any changes made by the hospital community pharmacy.

## 4. Discussion

The pre- and post-discharge interventions “patient education,” “discharge planning,” and “communication to primary care provider (PCP)” are commonly recommended by guidelines [26,27,28,29,30,31,32,33,34,35,36,37,38,39,40,41]. For the intervention: “patient education,” there is evidence, reported in a Cochrane review, for effectiveness in improved knowledge and satisfaction [23]. In Swiss hospitals, these interventions are frequently performed by different healthcare professionals. Our survey showed that “patient counselling on their medication,” “communication of medication changes to PCP,” “patient instructions,” and “in-depth patient counselling on their medication,” which were part of the classified interventions, were implemented most frequently. However, while pharmacists routinely counsel at-risk patient groups on their medication at hospital discharge in 44% of American hospitals [42], only one Swiss hospital routinely involved pharmacists in patient education at discharge. Studies have shown that pharmacist-led medication counselling to older patients at hospital discharge in combination with other interventions can significantly reduce hospital readmissions for all patients [43] or specific patient groups [44], reduce visits to the emergency department [45], and improve medication adherence [44] compared to standard care where the elements were conducted by a physician or nurse or not conducted at all.

Regarding the communication of medication changes, pharmacists were involved in this task only in two hospitals daily and in five hospitals less frequently. In comparison, 39% of participants of a European-wide survey indicated that, at transitions of care, pharmacists contribute to the transfer of information on medicines [46]. Studies have shown that pharmacists provide more comprehensive information on medication changes (e.g., new or stopped medication, changed dose) to general practitioners than physicians [47] and that community pharmacies need to contact hospital physicians less often when pharmacists add information on medication changes to discharge prescriptions [16]. A reduction of interruptions by such inquiries may increase the efficiency of hospital physicians and may possibly contribute to patient safety [16]. One hospital community pharmacy that participated in our study communicated changes to patients’ regular community pharmacies on a daily basis. A study has shown that community pharmacies appreciate receiving discharge prescriptions (including information on new and stopped medications) before patients come to the pharmacy [48]. They feel that, this way, they have more time to prepare the prescriptions and resolve discrepancies. Additionally, the community pharmacies in the mentioned study received the discharge summary, which was also considered to be helpful by most pharmacists [48]. It would therefore be beneficial for the quality of the communication of medication changes to involve pharmacists more often in this process. It would also be helpful to communicate changes not only to the patient’s general practitioner, but also to their regular community pharmacy.

Overall, at hospital discharge, pharmacists were not regularly involved in interventions to support patients with their medication management. This may be because of a lack of resources. In Swiss hospitals, one will find only 1.12 full-time equivalents for pharmacists per 100 beds [12]. In addition to the resources, patient-centred services require adequately trained pharmacists to perform these interventions. However, Swiss hospitals only offer 18.5 training positions for the certificate of proficiency in clinical pharmacy, a postgraduate education program focusing on patient-oriented pharmaceutical activities [12]. Another reason may be that, although medication safety is gaining importance in the Swiss Federal Office of Public Health, there are no legally binding recommendations for Swiss hospitals to implement such services [49].

One strategy of improving patient safety at hospital discharge could be a community pharmacy in or close by the hospital accessible to patients being discharged. Seventeen of the participating hospitals indicated to have such a community pharmacy or a counter in the hospital pharmacy. The main role of these pharmacies seemed to be the initial medication supply to patients at discharge. However, they were not often involved in tasks such as medication reconciliation or communication with patients’ community pharmacies. Community pharmacies in close collaboration with the hospital, especially with full access to the hospitals’ patient records, would be well-positioned for such services. Pharmacist-led medication reconciliation at hospital transitions has been shown to reduce medication discrepancies, all-cause readmissions, and all-cause emergency department visits [6,50].

One hospital implemented a medication self-management training program. The ability to take medication correctly after discharge is important, and helping patients to learn to do so can have a favorable impact on health outcomes. In the last few years, patient self-management has gained awareness [51], and, in a systematic review, self-management education or coaching showed a significant reduction in hospital readmissions [10]. The Society of Hospital Pharmacists of Australia recommended that patients who are enrolled in self-administration of medication programs should be counselled by a pharmacist (e.g., indication, dosage, and storage requirements) [52]. However, this service may be more suited for patients with a longer hospital stay to allow sufficient time for the observation and evaluation of included patients (as was the case in the hospital that implemented this service).

Of the six hospitals where the interviews took place, pharmacists were involved at hospital admission in only one hospital. In this hospital, pharmacists conducted a medication reconciliation and a medication review for patients with planned admission. Comprehensive involvement of pharmacists throughout the hospital stay has been shown to have positive effects on readmission within 30 days and 180 days [43]. Lack of resources may be a barrier for the implementation of medication reconciliation and medication review. A risk score could help select patients who benefit most from such services, e.g., patients with a risk for drug-related problems [53].

One strength of our study was the high return rate (73.3%) of the survey, which resulted in a representative sample for this study. In addition to the information obtained through the questionnaire, the follow-up interviews with selected hospitals gave a more detailed insight into pharmacists’ involvement in the hospitals’ processes. However, both the survey and the interviews relied on self-reporting, which allowed for reporting bias. It is also possible that interventions were implemented without pharmacists’ knowledge. This could have resulted in underreporting of some interventions.

## 5. Conclusions

In conclusion, interventions such as patient counselling and communication to primary care providers are widely recommended in guidelines. While Swiss hospitals mostly implemented these interventions, pharmacists were rarely involved. Some hospitals chose to support patients being discharged through a hospital community pharmacy, which performed different interventions. As studies have shown that involving pharmacists at hospital discharge can reduce readmissions, the results presented in this study should encourage hospitals to expand the involvement of pharmacists in medication management at hospital discharge.

## Figures and Tables

**Table 1:** Interventions covered in the survey with a brief description; taxonomy adapted from Hansen et al. [17].

Intervention	Description
Patient education	Patient counselling on their medication (e.g., therapy duration/dosing) In-depth patient counselling on their medication (e.g., effect/benefit, therapy goal/side effects) Patient instructions (e.g., for inhalation devices/prefilled syringe) Patient counselling on red flags (symptoms that indicate a worsening of condition/medication intolerance) Patient counselling on medication prescribed before hospital admission
Discharge planning	Organization of medicines (e.g., contact with patients’ community pharmacies/pill dispensers/reimbursement) Organization of rehabilitation/home care
Appointment scheduled before discharge	Assuring follow-up care (e.g., a follow-up appointment with the treating physician)
Timely follow-up	Follow-up care by a case manager
Communication to PCP	Medication changes are communicated to at least one healthcare professional (e.g., practitioner, community pharmacy) or institution (e.g., home care, nursing home)
Follow-up telephone call	Follow-up telephone call with discharged patients
Patient hotline	Hospital or hospital community pharmacy ^1^ offers a patient hotline for medication related questions

^1^ A hospital community pharmacy is a community pharmacy with full access to the hospital’s patient records. PCP = primary care provider.

**Table 2:** Number of hospitals that implemented interventions^1^ to support patients at hospital discharge (*n* = 44) and evidence promoting the implementation of these interventions.

	Intervention	Activities in Swiss Hospitals	Frequency, *n*	Evidence in Cochrane Reviews	Guidelines
Pre-discharge	Patient education	Patient counselling on their medication (e.g., therapy duration/dosing)	40	+ [23]	+ [26,27,28,29,30,31,33,34,35,36,37,38,39,40,41]
		In-depth patient counselling on their medication (e.g., effect/benefit, therapy goal/side effects)	32		
		Patient instructions (e.g., for inhalation devices/prefilled syringe)	33		
		Patient counselling on red flags (symptoms that indicate a worsening of condition/medication intolerance)	26		
		Patient counselling on medication prescribed before hospital admission	19		
	Discharge planning	Organization of medicines (e.g., contact with patients’ community pharmacies/pill dispensers/reimbursement)	9 *	+/0 [24]	+ [26,28,29,30,31,32,33,34,35,36,37,38,39,40]
		Organization of rehabilitation/home care	20 *		
	Appointment scheduled before discharge	Assuring follow-up care (e.g., a follow-up appointment with the treating physician)	15 *	?	+ [28,32,33,35,36,38]
Post-discharge	Timely follow-up	Follow-up care by a case manager	7 *	?	+ [28,30,34,35,39,40,42,43]
	Communication to PCP	Medication changes are communicated to at least one healthcare professional (e.g., practitioner, community pharmacy) or institution (e.g., home care, nursing home)	34	?	+ [26,27,28,29,30,31,33,35,37,38,39,41]
	Follow-up telephone call	Follow-up telephone call with discharged patients	2	+/0 [25]	+ [28,30,37,38]
	Patient hotline	Hospital or hospital community pharmacy offers a patient hotline for medication related questions	10	?	+ [32,35,36,38,40]

+ = there is evidence of effectiveness in the Cochrane reviews for the intervention or the intervention is recommended by the guidelines. +/0 = there is conflicting evidence of effectiveness in the Cochrane reviews for the intervention. ? = no data was found for the intervention. * This question was only answered by pharmacists that indicated to have a case manager in their hospital (*n* = 24). PCP = primary care provider. ^1^ Interventions were conducted by any healthcare professional (e.g., physician, pharmacist, nurse).

**Table 3:** Overview of implemented models to support patients throughout the hospital stay in interviewed hospitals (*n* = 6).

Hospital	Hospital Admission	Hospital Stay	Hospital Discharge	Follow-Up
	Interventions at Admission	Interventions during Hospital Stay	Pre-Discharge Interventions	Post-Discharge Interventions
Hospital 1	- Medication reconciliation - Medication review	- Interprofessional ward rounds (medical ward)	Hospital community pharmacy *#: - Medication reconciliation - Medication review - Patient education	Hospital community pharmacy *#: - Patient hotline
Hospital 2	None	- Medication self-management training program # - Group training for patients on different topics (e.g., bowel management)	Hospital community pharmacy *#: - Medication reconciliation - Patient education	Hospital community pharmacy *#: - Patient hotline
Hospital 3	None	- Interprofessional ward rounds (oncology ward) - Medication review (oncology and palliative ward)	None	None
Hospital 4	None	None	Hospital community pharmacy *#: - Medication reconciliation - Patient education	Hospital community pharmacy *#: - Patient hotline
Hospital 5	None	- Medication review (medical ward)	Hospital pharmacy#: - Medication reconciliation - Medication review	None
Hospital 6	None	- Interprofessional ward round (medical ward)	Hospital community pharmacy *#: - Patient education	Hospital community pharmacy *#: - Patient hotline

* We defined a hospital community pharmacy as a pharmacy open to patients discharged from the hospital with full access to the hospital’s patient records. # Interventions are further detailed in the text below.

## Data Availability

The data presented in this study are available on reasonable request from the corresponding author. The data are not publicly available due to confidentiality issues (e.g., interviews not anonymous).

## Appendix A. Cochrane Search

#1 (Continu* NEXT “of Patient Care” OR Aftercare OR Discharg* OR Handoff OR Transfer OR “Retention in Care” OR Trans* NEXT “to Adult Care” OR Trans* NEXT Care OR handover* OR “transitional care” OR “seamless care” OR patient* NEXT transition* OR “transmural care”)(:ti,ab)

#2 MeSH descriptor: [Continuity of Patient Care] 2 tree(s) exploded

#3 Hospital

#4 (#1 OR #2) AND #3

#5 #4 AND “Impact of medication reconciliation for improving transitions of care”

#6 MeSH descriptor: [Aftercare] this term only

#7 MeSH descriptor: [Patient Discharge] this term only

#8 MeSH descriptor: [Patient Handoff] this term only

#9 MeSH descriptor: [Patient Transfer] this term only

#10 MeSH descriptor: [Retention in Care] this term only

#11 MeSH descriptor: [Transition to Adult Care] this term only

#12 MeSH descriptor: [Transitional Care] this term only

#13 MeSH descriptor: [Continuity of Patient Care] this term only

#14 (#1 OR #2 OR #6 OR #7 OR #8 OR #9 OR #10 OR #11 OR #12 OR #13) AND #3

## Appendix B. Guideline Search

- Clinical guideline and hospital discharge and *
- Guideline * continuity OR aftercare OR handoff OR transfer OR handover OR “transitional OR care” OR “seamless OR care” OR transition OR “hospital discharge” -covid-19

* Interchangeable terms: England, Wales, Scotland, Northern Ireland, Ireland, Canada, United States of America, Australia, New Zealand.

## References

[1] Berry J.G., Gay J.C., Maddox K.J., Coleman E.A., Bucholz E.M., O’Neill M.R., Blaine K., Hall M. (2018). Age trends in 30 day hospital readmissions: US national retrospective analysis. BMJ.

[2] El Morabet N., Uitvlugt E.B., van den Bemt B.J., van den Bemt P.M., Janssen M.J., Karapinar-Çarkit F. (2018). Prevalence and Preventability of Drug-Related Hospital Readmissions: A Systematic Review. J. Am. Geriatr. Soc..

[3] Hawes E.M., Pinelli N.R., Sanders K.A., Lipshutz A.M., Tong G., Sievers L.S., Chao S., Gwynne M. (2018). Post-Hospital Discharge Care: A Retrospective Cohort Study Exploring the Value of Pharmacist-Enhanced Care and Describing Medication-Related Problems. North Carol. Med. J..

[4] Kripalani S., Roumie C.L., Dalal A.K., Cawthon C., Businger A., Eden S.K., Shintani A., Sponsler K.C., Harris L.J., Theobald C.N. (2012). Effect of a Pharmacist Intervention on Clinically Important Medication Errors After Hospital Discharge. Ann. Intern. Med..

[5] De Oliveira G.S., Castro-Alves L.J., Kendall M.C., McCarthy R. (2017). Effectiveness of Pharmacist Intervention to Reduce Medication Errors and Health-Care Resources Utilization After Transitions of Care. J. Patient Saf..

[6] Mekonnen A.B., McLachlan A.J., Brien J.-a.E. (2016). Effectiveness of pharmacist-led medication reconciliation programmes on clinical outcomes at hospital transitions: A systematic review and meta-analysis. BMJ Open.

[7] Daliri S., Hugtenburg J.G., Ter Riet G., van den Bemt B.J.F., Buurman B.M., Reimer W.J.M.S.O., Van Buul-Gast M.-C., Karapinar-Çarkit F. (2019). The effect of a pharmacy-led transitional care program on medication-related problems post-discharge: A before—After prospective study. PLoS ONE.

[8] Bonetti A.F., Reis W.C., Lombardi N.F., Mendes A.M., Netto H.P., Rotta I., Fernandez-Llimos F., Pontarolo R. (2018). Pharmacist-led discharge medication counselling: A scoping review. J. Eval. Clin. Pract..

[9] Capiau A., Foubert K., Van Der Linden L., Walgraeve K., Hias J., Spinewine A., Sennesael A.-L., Petrovic M., Somers A., Belgian Society for Gerontology and Geriatrics (BSGG) (2020). Medication Counselling in Older Patients Prior to Hospital Discharge: A Systematic Review. Drugs Aging.

[10] Tomlinson J., Cheong V.-L., Fylan B., Silcock J., Smith H., Karban K., Blenkinsopp A. (2020). Successful care transitions for older people: A systematic review and meta-analysis of the effects of interventions that support medication continuity. Age Ageing.

[11] Leppin A.L., Gionfriddo M.R., Kessler M., Brito J.P., Mair F.S., Gallacher K., Wang Z., Erwin P.J., Sylvester T., Boehmer K. (2014). Preventing 30-day hospital readmissions: A systematic review and meta-analysis of randomized trials. JAMA Intern. Med..

[12] Studer H., Boeni F., Messerli M., Hersberger K.E., Lampert M.L. (2020). Clinical pharmacy activities in Swiss hospitals: How have they evolved from 2013 to 2017?. Pharmacy.

[13] Bundesgesetz über Arzneimittel und Medizinprodukte. https://www.admin.ch/ch/d/sr/8/812.21.de.pdf.

[14] Swiss Association of Public Health Administration and Hospital Pharmacists Berufsbild des Spitalapothekers und Leitbild für Seine Weiterbildung. https://www.gsasa.ch/deliver.cfm?f=0CD89DA59212A7CBAEDB92D04866B8AB822D41B39CAE138ABE7A989FD2DB9A692B80BC5080BEBBCC6E97CABFB3488583E3A496F8AB8ED38C9B9F47BFBA04939D4D0AA14FBA9BB7ABAD1792ABEA5CCF75ED56AEBF87BB575C4E939F8DFE5E26BD5BDB7205173B05ACDFCBF495A8FC&type=.pdf.

[15] pharmaSuisse Weiterbildungsprogramm zum Fähigkeitsausweis FPH in klinischer Pharmazie. https://www.gsasa.ch/de/bildung-de/fph-klinische-pharmazie/weiterbildungsprogramm/?oid=10149&lang=de.

[16] Bruhwiler L.D., Beeler P.E., Boni F., Giger R., Wiedemeier P.G., Hersberger K.E., Lutters M. (2019). A RCT evaluating a pragmatic in-hospital service to increase the quality of discharge prescriptions. Int. J. Qual. Health Care.

[17] Hansen L.O., Young R.S., Hinami K., Leung A., Williams M.V. (2011). Interventions to reduce 30-day rehospitalization: A systematic review. Ann. Intern. Med..

[18] Coleman E.A., Parry C., Chalmers S., Min S.J. (2006). The care transitions intervention: Results of a randomized controlled trial. Arch. Intern. Med..

[19] Bobay K., Bahr S.J., Weiss M.E., Hughes R., Costa L. (2015). Models of discharge care in Magnet(R) hospitals. J. Nurs. Adm..

[20] Pedersen C.A., Schneider P.J., Scheckelhoff D.J. (2015). ASHP national survey of pharmacy practice in hospital settings: Dispensing and administration--2014. Am. J. Health-Syst. Pharm..

[21] Cawthon C., Walia S., Osborn C.Y., Niesner K.J., Schnipper J.L., Kripalani S. (2012). Improving care transitions: The patient perspective. J. Health Commun..

[22] Pedersen C.A., Schneider P.J., Scheckelhoff D.J. (2013). ASHP national survey of pharmacy practice in hospital settings: Monitoring and patient education--2012. Am. J. Health-Syst. Pharm..

[23] Johnson A., Sandford J., Tyndall J. (2003). Written and verbal information versus verbal information only for patients being discharged from acute hospital settings to home. Cochrane Database Syst. Rev..

[24] Goncalves-Bradley D.C., Lannin N.A., Clemson L.M., Cameron I.D., Shepperd S. (2016). Discharge planning from hospital. Cochrane Database Syst. Rev..

[25] Mistiaen P., Poot E. (2006). Telephone follow-up, initiated by a hospital-based health professional, for postdischarge problems in patients discharged from hospital to home. Cochrane Database Syst. Rev..

[26] National Institute for Health and Care Exellence Transition between Inpatient Hospital Settings and Community or Care Home Settings for Adults with Social Care Needs. https://www.nice.org.uk/guidance/ng27/evidence/full-guideline-pdf-2185185565.

[27] Royal Pharmaceutical Society Keeping Patients Safe When They Transfer between Care Providers—Getting the Medicines Right. https://www.rpharms.com/Portals/0/RPS%20document%20library/Open%20access/Publications/Keeping%20patients%20safe%20transfer%20of%20care%20report.pdf.

[28] Alper E., O’Malley T.A., Greenwald J. UptoDate: Hospital Discharge and Readmission. https://www.uptodate.com/contents/hospital-discharge-and-readmission#H19.

[29] Head of Clinical Services–Community Hospitals and Transitional Care, Lincolnshire Community Health Services Admission, Discharge and Transfer Policy for Community Hospitals. https://www.lincolnshirecommunityhealthservices.nhs.uk/application/files/7115/4221/1756/P_CS_06_Admission_Discharge_and_Transfer_Policy.pdf.

[30] Health Service Executive National Integrated Care Advisory Group Integrated Care Guidance: A Practical Guide to Discharge and Transfer from Hospital. https://www.hse.ie/eng/about/who/qid/resourcespublications/nationalintegratedcareguidance.pdf.

[31] Aneurin Bevan University Health Board Discharge Policy. http://www.wales.nhs.uk/sitesplus/documents/866/FOI%2017076%20Enclosure.pdf.

[32] Head of Capacity & Corporate Nursing Policy & Procedure for Discharge Practices. https://hgs.uhb.nhs.uk/wp-content/uploads/Discharge-Practices-Policy.pdf.

[33] Change Agent Team, National Leadership and Innovation Agency for Healthcare Passing the Baton: A Practical Guide to Effective Discharge Planning. https://www.adss.cymru/en/blog/post/passing-the-baton-a-practical-guide-to-effective-discharge-planning.

[34] Agency for Healthcare Research and Quality Care Transitions from Hospital to Home: IDEAL Discharge Planning. https://www.ahrq.gov/sites/default/files/wysiwyg/professionals/systems/hospital/engagingfamilies/strategy4/Strat4_Implement_Hndbook_508_v2.pdf.

[35] Registered Nurses’ Association of Ontario Care Transitions—Clinical Best Practice Guidelines. https://rnao.ca/sites/rnao-ca/files/Care_Transitions_BPG.pdf.

[36] Health Quality Ontario Adopting a Common Approach to Transitional Care Planning: Helping Health Links Improve Transitions and Coordination of Care. http://www.hqontario.ca/Portals/0/documents/qi/health-links/bp-improve-package-traditional-care-planning-en.pdf.

[37] The Society of Hospital Pharmacists of Australia Compiled Quick Guides SHPA. https://www.shpa.org.au/sites/default/files/uploaded-content/website-content/SOP/quick_guides_e-book_2013.pdf.

[38] The Society of Hospital Pharmacists of Australia Chapter 6: Facilitating Continuity of Medication Management on Transition between Care Settings. https://www.shpa.org.au/sites/default/files/uploaded-content/website-content/SOP/sop_clinical_pharmacy_s26-s28_chapter6.pdf.

[39] New Zealand HealthCare Pharmacists’ Association Clinical Pharmacy Guidelines. https://www.google.com/url?sa=t&rct=j&q=&esrc=s&source=web&cd=&cad=rja&uact=8&ved=2ahUKEwiFvaujlvDrAhVKDuwKHbs1Dmc4ChAWMAV6BAgGEAE&url=http%3A%2F%2Fwww.nzhpa.org.nz%2Fmedia%2F1379%2Fclinguide_07.pdf&usg=AOvVaw33_bwFGOQJ9MR5NxKT_JW6.

[40] Health & Social Care Joint Unit and Change Agents Team Discharge from Hospital: Pathway, Process and Practice. http://www.wales.nhs.uk/sitesplus/documents/829/DoH%20-%20Discharge%20Pathway%202003.PDF.

[41] Department of Health, Government of South Australia Continuity in Medication Management—A Handbook for South Australian Hospitals. https://www.sahealth.sa.gov.au/wps/wcm/connect/e055bd8044fd8fc2aff7efcfa5ded0ab/Pharmaceutical+Reform+Handbook+V7_Print+version.pdf?MOD=AJPERES&ampCACHEID=ROOTWORKSPACE-e055bd8044fd8fc2aff7efcfa5ded0ab-n5iXGmp.

[42] Pedersen C.A., Schneider P.J., Ganio M.C., Scheckelhoff D.J. (2019). ASHP national survey of pharmacy practice in hospital settings: Monitoring and patient education—2018. Am. J. Health-Syst. Pharm..

[43] Ravn-Nielsen L.V., Duckert M.L., Lund M.L., Henriksen J.P., Nielsen M.L., Eriksen C.S., Buck T.C., Pottegard A., Hansen M.R., Hallas J. (2018). Effect of an in-hospital multifaceted clinical pharmacist intervention on the risk of readmission: A randomized clinical trial. JAMA Intern. Med..

[44] Bonetti A., Bagatim B., Mendes A., Rotta I., Reis R., Fávero M., Fernandez-Llimós F., Pontarolo R. (2018). Impact of discharge medication counseling in the cardiology unit of a tertiary hospital in Brazil: A randomized controlled trial. Clinics.

[45] Gillespie U., Alassaad A., Henrohn D., Garmo H., Hammarlund-Udenaes M., Toss H., Kettis-Lindblad A., Melhus H., Morlin C. (2009). A comprehensive pharmacist intervention to reduce morbidity in patients 80 years or older: A randomized controlled trial. Arch Intern. Med..

[46] Horák P., Gibbons N., Sýkora J., Batista A., Underhill J. (2017). EAHP statements survey 2016: Sections 1, 3 and 4 of the European Statements of Hospital Pharmacy. Eur. J. Hosp. Pharm..

[47] Ooi C.E., Rofe O., Vienet M., Elliott R.A. (2017). Improving communication of medication changes using a pharmacist-prepared discharge medication management summary. Int. J. Clin. Pharm..

[48] McCarthy L.M., Li S., Fernandes O., Cameron K., Lui P., Wong G., Pariser P., Farrell J., Luke M.J., Guilcher S.J.T. (2019). Enhanced communication between inpatient and community pharmacists to optimize medication management during transitions of care. J. Am. Pharm. Assoc..

[49] Vincent C., Staines A. Enhancing the Quality and Safety of Swiss Healthcare. https://www.bag.admin.ch/bag/en/home/versicherungen/krankenversicherung/krankenversicherung-qualitaetssicherung.html.

[50] Mekonnen A.B., McLachlan A.J., Brien J.A. (2016). Pharmacy-led medication reconciliation programmes at hospital transitions: A systematic review and meta-analysis. J. Clin. Pharm. Ther..

[51] Vanwesemael T., Boussery K., Dilles T. (2020). Self-administration of medication in hospital: A literature review. Nurs. Sci. Q..

[52] Davis A., Muir P., Allardice J.-A., Clark K., Groves J., Molenaar M., Robson G. (2002). SHPA guidelines for self-administration of medication in hospitals and residential care facilities. J. Pharm. Pract. Res..

[53] Stampfli D., Boeni F., Gerber A., Battig V.A.D., Weidmann R., Hersberger K.E., Lampert M.L. (2018). Assessing the ability of the Drug-Associated Risk Tool (DART) questionnaire to stratify hospitalised older patients according to their risk of drug-related problems: A cross-sectional validation study. BMJ Open.

